# The Uptake of Prevention of Mother-to-Child HIV Transmission Programs in China: A Systematic Review and Meta-Analysis

**DOI:** 10.1371/journal.pone.0135068

**Published:** 2015-08-26

**Authors:** Zhaohui Huang, Meihua Jin, Huan Zhou, Zhengquan Dong, Sichao Zhang, Jiankang Han, Keqin Fu, Jianli Wu, Shudong Wu, Huarong Du, Zhongrong Yang

**Affiliations:** 1 Anhui Provincial Family Planning Institute of Science and Technology, Hefei 230031, Anhui Province, China; 2 Huzhou Center for Disease Control and Prevention, Huzhou 313000, Zhejiang Province, China; 3 Health management teaching and research section, the first affiliated hospital of Bengbu medical college, Bengbu, Anhui 233000, P.R. China; Institute of Infection and Global Health, UNITED KINGDOM

## Abstract

**Background:**

No systematic review of prevention of mother to child transmission (PMTCT) in China has been performed. We aimed to estimate the uptake of PMTCT programs services in China.

**Methods:**

We searched MEDLINE, EMBASE, Cochrane Central Register of Controlled Trials, China National Knowledge Infrastructure, Chinese Biomedical Literature Database, and Wanfang (Chinese) to identify research studies. Only descriptive epidemiological studies were eligible for this study.

**Results:**

A total of 57 eligible cross-section studies were finally included. We estimated that the mean HIV-positive rate of exposed infants was 4.4% (95% CI = 3.2–5.5), and more than 33% of exposed infants had not undergone HIV diagnostic testing. The percentage of initiating antiretroviral therapy (ART) in HIV-positive women was 71.0% (95% CI = 66.3–75.8), and that for initiating antiretroviral prophylaxis (ARP) in exposed infants was 78.3% (95% CI = 74.9–81.8); also, 31.3% (95% CI = 15.5–47.0) of women with HIV and < 1% of exposed infants received the combination of three antiretroviral drugs. There were bigger gap of uptake of PMTCT programs between income levels, and cities with a low income level had a higher percentage of initiating ART in HIV-positive women (80%) and ARP in exposed infants (85%) compared to cities with high-middle income (57% and 65%, respectively) (P<0.05).

**Conclusions:**

This paper highlights the need to further scale up PMTCT services in China, especially in regions with the lowest coverage, so that more women can access and utilize them. However, some estimated outcome should be interpreted with caution due to the high level of heterogeneity and the small number of studies.

## Introduction

Globally, approximately 34 million people were HIV-positive in 2011, including 3.3 million children younger than 15 years old, and of these children, 330,000 became newly infected with HIV in 2011, over 90% of them by vertical transmission[1,2]. Prompt implementation of effective prevention of mother-to-child transmission(PMTCT) of HIV programs helped prevent more than 800,000 children from becoming newly infected between 2005 and the end of 2012[3].Mother-to-child transmission (MTCT) rates in the developed regions have decreased to 1% or less due to widespread scale up of PMTCT of HIV programs[4]. The first case of an infant infected with HIV by vertical transmission in China was reported in 1995; afterwards, the MTCT rate rapidly increased to 0.4% in 2002. The Chinese ministry of health launched a PMTCT program in China in 2003 [5]. In 2004, the Chinese ministry of health issued a guideline on PMCTC that included HIV counseling and testing services, highly active antiretroviral therapy (HAART), elective cesarean section, and avoiding breastfeeding[6]. It is estimated that more than 780,000 people were living with HIV at the end of 2011 in China; 28.6% were women and 1.1% were children infected through MTCT[7].

Over the past 10 years, substantial progress has been made in the implementation of PMTCT interventions in China. PMTCT strategic vision 2010–2015, published by the World Health Organization (WHO), emphasized tracking program performance and its impact on the MTCT rates and on maternal and child health[8]. However, studies that were conducted to track the progress of the programs were insufficient, and a systematic review on the uptake of these programs has not been performed in China.

Only descriptive epidemiological studies that provide data regarding PMTCT interventions programs were eligible for this study. The goal of this systematic review is to provide an overview and pooled prevalence estimate of HIV-positive among pregnant women and exposed infants in China. We also provide an overview and pooled uptake estimate of some important PMTCT interventions programs including voluntary HIV counseling and testing, antiretroviral therapy and prophylaxis in pregnant women and infants, HIV-diagnosis of the exposed infants, termination of pregnancy, elective cesarean section and artificial feeding in China. In addition, we also provide a simple analysis of several trends and distributions available from the data in order to provide more insight into better treatment and control of MTCT of HIV in China.

## Methods

### Search Strategy and Selection Criteria

We searched Cochrane Central Register of Controlled Trials (CENTRAL), MEDLINE, EMBASE, China National Knowledge Infrastructure (CNKI), Chinese Biomedical Literature Database (CBM), and Wanfang (Chinese) to identify research studies that described the uptake of PMTCT programs in China.Details on search terms can be found in the S1 Appendix. The same search strategies were used with each database.We placed no language restrictions on the searches or search results. Additional strategies included hand searches of journals that were not indexed in the electronic sources, web-based searches, and screening of reference lists of retrieved studies for additional potentially relevant articles.

Only descriptive epidemiological studies were eligible for this study. When a study reported the results from different subpopulations, we treated them independently. We excluded meetings, literature reviews, discussions, editorials, research overviews, book reviews, letters, and news articles. We excluded qualitative studies, modeling studies, studies where uptake of PMTCT was assessed by interview, and cost-effectiveness studies. We excluded studies that did not provide useable data. Studies with fewer than 30 participants were excluded to improve the efficiency of the analysis.

**Fig 1:**
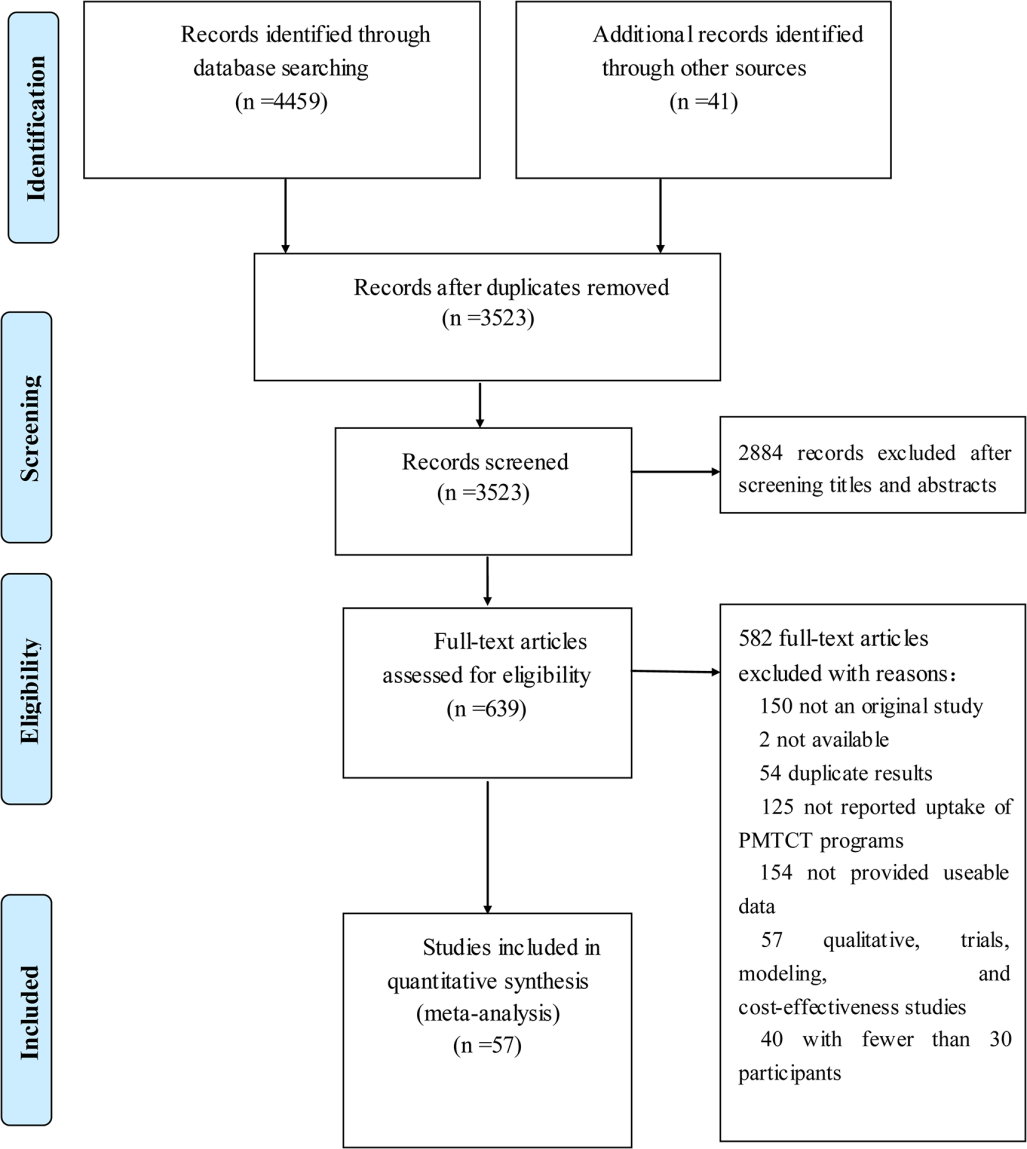
Prisma 2009 Flow diagram literature search and study selection. PRISMA diagram showing the different steps of systematic review, starting from literature search to study selection and exclusion. At each step, the reasons for exclusion are indicated.doi:10.1371/journal.pone.0052562.g001).

Two of the authors (MJ and HZ)independently screened the titles and abstracts of all identified studies. Studies that appeared to be relevant were selected, and the same two reviewers (MJ and HZ)independently assessed the full-text versions. Disagreements were resolved by consensus or the involvement of a third reviewer(ZH). Fig 1 shows the flowchart for selecting articles[9–65].

### Data Collection

We developed and modified a data abstraction form after a training exercise for investigators. We extracted the following data from the eligible studies: characteristics of the studies (years, design, ART regimen, and regions), participants (age, sex, and the status of HIV infection), and primary and second outcomes. We also gathered data on potential explanatory variables (i.e., variables that might explain the variance in uptake of PMTCT; Table 1). We also obtained data from the website of the Chinese administrative region; these data were used to categorize regions into regions (EasternChina, south-central China, northChina, northwestChina, southwestern China, northeastChina, and special district of Taiwan, Hongkong, and Macao) and gross domestic product in $US per head in 2013 (low income (<5520), low-middle income (5635–6750), high-middle income (6892–9961), and high income (>10915))[66,67].

For each study, one reviewer(ZD) extracted the data, a second reviewer (SZ)checked the accuracy, and a third reviewer (JH)evaluated the data for disagreements.

**Table 1:** Characteristics of Studies Included in the Meta-Analysis.

Study, year	Province	Region	Income level$	Study design	Sample	Outcomes#	Scores of study quality&
Wang Q, 2006	Sinkiang, Yunnan	Trans-reginoal	Low income	cross-sectional	774	2, 3	6
Zhang SW, 2012	Anhui	Eastern China	Low income	cross-sectional	642059	1,2,3,	6
Wang QF, 2011	Yunnan	Southwestern China	Low income	cross-sectional	78681	1,3,4,5,10	4
Tan YM, 2013	Guangxi	South-central China	Low income	cross-sectional	363817	1,2,3	4
Yang M, 2011	Guizhou	Southwestern China	Low income	cross-sectional	214	4, 10	5
Ma 5, 2010	Henan	South-central China	Low income	cross-sectional	36	4,5,10	4
Wu M, 2011	Hunan	South-central China	Low-middle income	cross-sectional	1223557	1, 3, 4,6,7, 10	5
Du M, 2006	Yunnan	Southwestern China	Low income	cross-sectional	9145	1,2,3	4
Ma Q, 2012	Yunnan	Southwestern China	Low income	cross-sectional	204687	1,2,3,5,6,7,9	4
Huang S, 2013	Guangdong	South-central China	High-middle income	cross-sectional	88	5	5
Cheng WM, 2009	Henan	South-central China	Low income	cross-sectional	363491	1,3,4,5,7, 9	8
Li T, 2012	Sichuan	Southwestern China	Low income	cross-sectional	152	4,5,6,7, 8, 9, 10	6
Wang Q,2011	Yunnan	Southwestern China	Low income	cross-sectional	29095	1,3, 4,5, 7, 9, 10	7
Chen Y, 2013	Sinkiang	Northwest China	Low-middle income	cross-sectional	326030	1, 3,9	4
Bai Y, 2012	Guangxi	South-central China	Low-middle income	cross-sectional	50043	1,3,4,5,7,10	7
Cheng WM, 2006	Henan	South-central China	Low income	cross-sectional	31	4,5,7,9,10	4
Chen ZY, 2010	Henan	South-central China	Low income	cross-sectional	773928	1,3,5	6
Lu QC, 2008	Henan	South-central China	Low income	cross-sectional	171490	1,5,7,8, 9,10	6
Sun DY, 2008	Henan	South-central China	Low income	cross-sectional	763514	1,4,5,7, 9, 10	7
Wang XY, 2013	Sichuan, Yunnan, Sinkiang,	Trans-reginoal	Low income	cross-sectional	41586	1,3	8
Dai GH, 2010	Hubei	South-central China	High-middle income	cross-sectional	2288933	1, 4, 5, 6,7,8,9,10	7
Gong SY, 2007	Henan, Guangxi, Sinkiang,Yunnan	Trans-reginoal	Low income	cross-sectional	346	4,5,6,7,9	6
Hong Y, 2009	Yunnan	Southwestern China	Low income	cross-sectional	76994	2,3, 4,6,7,10	6
Li Y, 2012	Guizhou	Southwestern China	Low income	cross-sectional	325888	1,2,3,4, 5,6,7,8,9,10	7
Wang AL, 2006	Unclear	unclear	unclear	cross-sectional	40	4,5,7,10	4
Wang Q, 2012	Yunnan	Southwestern China	Low income	cross-sectional	90	4,7,8,9,10	6
Wang Q, 2009	Yunnan	Southwestern China	Low income	cross-sectional	15596	1,4, 5, 7, 8,9,10	6
Wang ZZ, 2007	Guangdong	South-central China	High-middle income	cross-sectional	746366	1,2,5,6, 7,10	8
Xiong YH, 2010	Yunnan	Southwestern China	Low income	cross-sectional	102068	1,4,5,7,10	5
Yang M, 2014	Guizhou	Southwestern China	Low income	cross-sectional	616783	1,4,8	8
Zhang XH, 2009	Zhejiang	Eastern China	High income	cross-sectional	140	4,5,7,10	6
Zhu XX, 2005	Yunnan	Southwestern China	Low income	cross-sectional	6440	1,4,5,7,10	5
Wang XY, 2010	Unclear	Trans-reginoal	Low income	cross-sectional	480	4,6,10	6
Wang Q, 2013	Henan, Guangxi, Sinkiang,Yunnan	Trans-reginoal	Low income	cross-sectional	1166	6	8
Wang WM, 2008	Henan	South-central China	Low income	cross-sectional	143	4,5,6,7,8,9,10	4
AilikaShawuli, 2013	Sinkiang	Northwest China	Low-middle income	cross-sectional	1303	4,10	6
Wang Q, 2013	Henan, Guangxi, Sinkiang,Yunnan,Gui	Trans-reginoal	Low income	Cohort study	1414	4,10	8
Jiang W, 2013	Guangxi	South-central China	Low income	cross-sectional	317	5	6
Wang YX, 2011	Guangdong	South-central China	High-middle income	cross-sectional	172669	1,4,5,7,8,9,10	6
Chen L, 2013	Guangxi	South-central China	Low income	cross-sectional	94454	1,3,4,5,10	6
Weng YQ, 2010	Guangxi	South-central China	Low income	cross-sectional	42626	1,2,3,4,5,7,10	8
Wen Y, 2011	Yunnan	Southwestern China	Low income	cross-sectional	57096	1,2,3,5,6,8,9,10	6
Cao YZ,2011	Guangdong	South-central China	High-middle income	cross-sectional	412525	3,5	5
Zhang HY, 2011	Chongqing	Southwestern China	High-middle income	cross-sectional	68	4,5,7,10	4
Zhou FR, 2010	Shandong	Eastern China	High-middle income	cross-sectional	118625	2,3	7
An FL, 2009	Henan	South-central China	Low income	cross-sectional	62028	1,5,7,9,10	6
Feng L, 2013	Chongqing	Southwestern China	High-middle income	cross-sectional	1789	1,3	8
Liang K, 2011	Hubei, Hebei, Shanxi, Sinkiang	Trans-reginoal	low-middle income	cross-sectional	421	5	5
Wang FK, 2009	Henan	South-central China	Low income	cross-sectional	339866	1,4,5,7,8,9	5
Fang LW, 2010	Cover 31 provinces	Trans-reginoal		cross-sectional	10360655	2,3,4,5,6,7,9,10	8
Luo XM, 2011	Hunan	South-central China	Low income	cross-sectional	98004	1,3	4
Sun LD, 2011	Yunnan	Southwestern China	Low income	cross-sectional	30101	1,3,4,5,8	6
Jia LQ, 2010	Yunnan	Southwestern China	Low income	cross-sectional	17425	1,2,3,4,5,7,8,9,10	6
Li B, 2013	Guangdong	South-central China	High-middle income	cross-sectional	108	4,6,7,10	8
Song JM, 2013	Guangdong	South-central China	High-middle income	cross-sectional	1843122	1,3,4,5,10	8
Lin AW, 2014	Hong Kong		High income	cross-sectional	489187	1,3,4,5,6	8
Zhao XH, 2013	Zhejiang	Eastern China	High income	cross-sectional	4359246	1,2,3,4,10	8

Note

# The primary outcomes for pregnant women were the following: (1) HIV-positive rate; (2) underwent voluntary HIV-counseling; (3) underwent voluntary HIV-testing; (4) initiating antiretroviral therapy (ART); (5) selecting termination of pregnancy; (6) elective cesarean section; and (7) artificialfeeding. The primary outcomes for children were the following: (8) HIV-positive rate (9) HIV diagnosis of the exposed infants between 12 and 18 months by PCR or antibody test; and (10) initiating ARP in exposed infants.

& Quality score assessed by Agency of Healthcare Research and Quality (AHRQ)

$ Income level is divided to high income (>10915), high-middle income (6892–9961), low-middle income, low income (<5520) according to GDP (in $US per head)

### Primary Outcomes

The primary outcomes for pregnant women were the following: (1) HIV-positive ratein the present pregnancy (including antenatal, intrapartum, and postpartum); (2) underwent voluntary HIV-counselingin the present pregnancy (including antenatal, intrapartum, and postpartum); (3) underwent voluntary HIV-testingin the present pregnancy (including antenatal, intrapartum, and postpartum); (4) initiating antiretroviral therapy (ART) from 28 weeks of pregnancy (5) selecting termination of pregnancy; (6) elective cesarean section; and (7) artificialfeeding. The primary outcomes for children were the following: (8) HIV-positive rate (9) HIV diagnosis of the exposed infants between 12 and 18 months by PCR or antibody test; and (10) initiating ARP in exposed infantsin the first 72 hours after delivery.

### Quality assessment

The quality of eligible literature was assessed according to the criteria of observational studies in recommended by Agency of Healthcare Research and Quality AHRQ included 11-items with a yes/no/unclear response option: the “Yes” would be scored “1”, “No” or “unclear” was scored “0”. Articles were scored as follows: low quality (0–3), moderate quality (4–7), high quality (8–11) [68].

### Statistical Analysis

We calculated the pooled rate or percentage with 95% CIs with a random effect model (DerSimonian–Laird’s method)[69]. We used the Q-statistic and I^2^ statistic to estimate the heterogeneity between studies, and we used “small,” “moderate” and “large” to describe values of 25%, 50% and 75% for the I^2^[70–71].

We investigated potential sources of heterogeneity with subgroup analyses. In subgroup analyses, we estimated the uptake of PMTCT according to regions (EasternChina, southChina, South-central China, northChina, northwest China, southwestern China, northeastChina, and special district of Taiwan Hongkong and Macao), per capita GDP (low income, low-middle income, high-middle income, and high income), pregnancy stage (premarital checkups, antenatal care, and at delivery), and antiretroviral therapy regimens (single regimen vs. combination regimens).

To establish the robustness of the outcome by sensitivity analyses, we applied a fixed effects model;used the trim-and-fill method; and excluded studies with a low number of participants [72].A funnel plot was used to explore the publication bias. Funnel-plot asymmetry was further assessed by the method of Begg’ test and the modified Egger’s linear regression test[73].We performed all analyses using the software STATA (version 11.0).

## Results

### Characteristics of Eligible Studies

We identified 4459papers from a database search, 28 papers through internet and hand searches, and 15 papers through checking reference lists. No unpublished data that met our inclusion criteria were identified. During the step of screening the abstracts, 2884 papers were excluded, leaving 639full text papers that were assessed for eligibility. We excluded 40 papers with fewer than 30 participants; in the end, a total of 57 papers fulfilled our inclusion criteria and were included in the meta-analysis (Fig 1). These 57 studies originated from 31 of 34 provinces in China. Table 1 presents the characteristics of every analysis outcome, 13 studies were of high quality, and other studies were of moderate quality.

### Estimated HIV-positive rate

**Fig 2:**
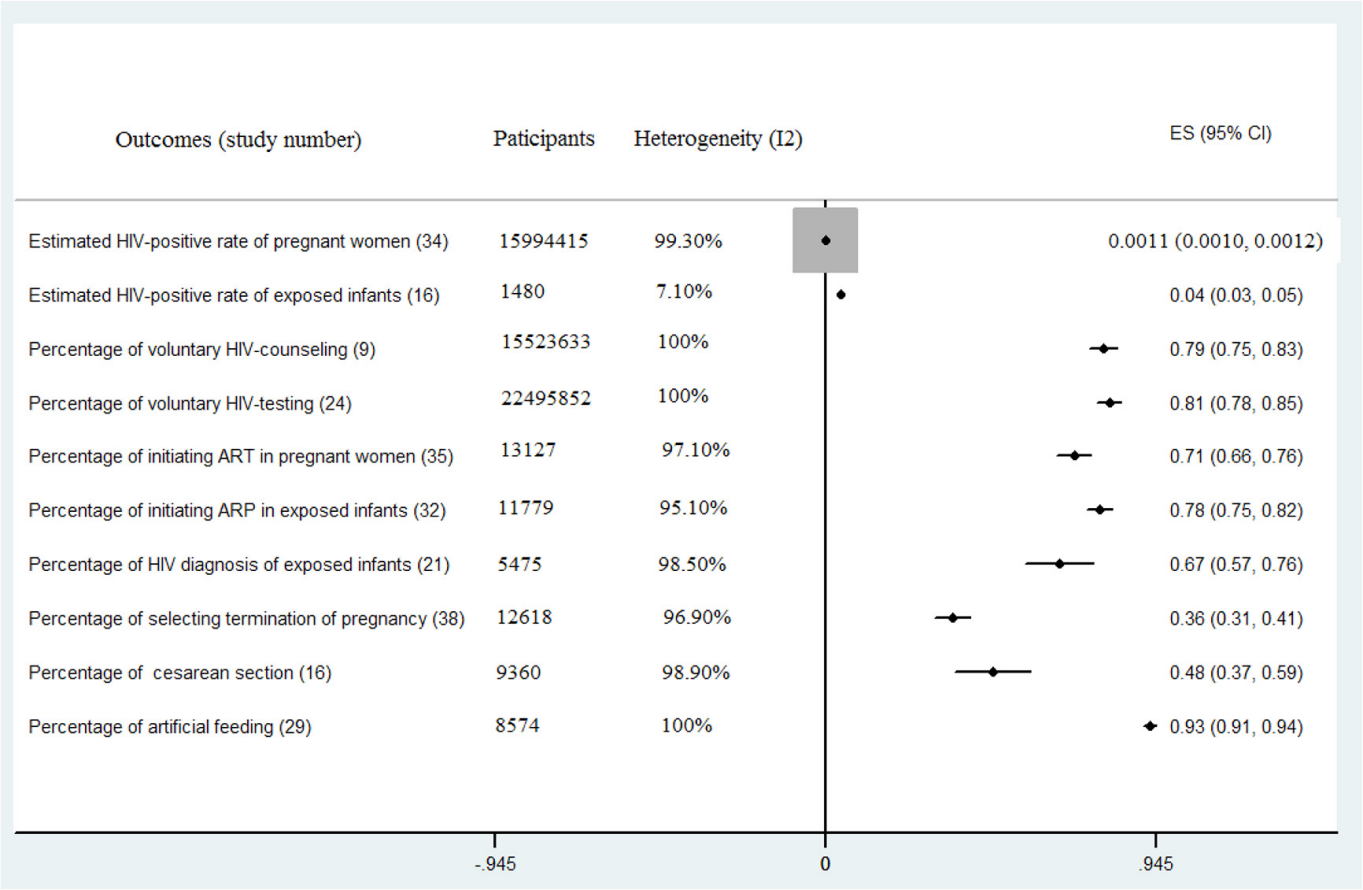
Meta-Analyses of Uptake of Prevention of Mother-to-Child-Transmission (PMTCT) in China. ART = antiretroviral therapy; ARP = antiretroviral prophylaxis; ES refer to rate /percentage.

Thirty-four studies, including 15,994,415 pregnant women, reported the HIV-positive rate of pregnant women (Fig 2). We estimated that the mean HIV-positive rate of pregnant women was 0.11% (95% CI = 0.1–0.12) with a high level of heterogeneity between the rate estimates (Q = 5049.43, P<0.001; I^2^ = 99.30%). Sixteen studies, including 1,480 infants, reported the HIV-positive rate of exposed infants. We estimated that the mean HIV-positive rate of exposed infants was 4.4% (95% CI = 3.2–5.5) with a low level of heterogeneity between the rate estimates (Q = 13.96, P = 0.602; I^2^ = 7.10%) (Fig 2 and Table 2).

**Table 2:** Egger’s linear regression test and Begg's test to measure the funnel plot asymmetric.

Outcome	Heterogeneity			Egg's test		Begg's test	
	Q	p	I^2^(%)	t	P	z	p
Estimated HIV-positive rate of pregnant women	5049.93	<0.001	99.3	6.63	0	2.98	0.003
Estimated HIV-positive rate of exposed infants	13.96	0.602	7.1	1.77	0.077	2.05	0.058
Percentage of voluntary HIV-counseling	320000	<0.001	100	-0.5	0.628	0.09	0.929
Percentage of voluntary HIV-testing	160000	<0.001	100	-1.25	0.222	-1.61	0.108
Percentage of initiating ART in HIV-positive women	1335.82	<0.001	97.1	-0.7	0.486	-2.25	0.025
Percentage of initiating ARP in exposed infants	617.39	<0.001	95.1	-2.58	0.031	-3	0.003
Percentage of HIV diagnosis of the exposed infants	970.78	<0.001	98.5	-1.67	0.096	-0.62	0.545
Percentage of selecting termination of pregnancy	12909.6	<0.001	96.9	1.68	0.101	0.94	0.346
Percentage of cesarean section	1370.5	<0.001	98.9	0.16	0.874	0.81	0.418
Percentage of artificial feeding	1255.5	<0.001	100	-0.94	0.358	-2.98	0.003

### Estimated Uptake of PMTCT Programs Services

The estimated percentages of voluntary HIV-counseling and HIV-testing of pregnant women were 79.3% (95% CI = 75.4–83.3) and 81.2% (95% CI = 77.8–84.6), respectively. The studies were very heterogeneous in both of the analyses (Fig 2). We identified 35 studies providing data for the percentage of initiating ART in HIV-positive women and 32 studies providing data for the percentage of initiating ARP in exposed infants. We estimated that the percentage of initiating ART in HIV-positive women was 71.0% (95% CI = 66.3–75.8) and initiating ARP in exposed infants was 78.3% (95% CI = 74.9–81.8); these estimates were also associated with a high level of heterogeneity (Fig 2).Other outcomes were also estimated, and the results are displayed in Fig 2.

### Subgroup Analyses of the Uptake of PMTCT Programs

Table 3 and Table 4 present the variation in the uptake of PMTCT programs according to years. No study on PMTCT programs in China was reported in China before 2004. The time trends for the HIV-positive rate of pregnant were reported in 34 studies from 2000–2012; the mean estimated rate was not significantly changed since 2000. However, time trends for the HIV-positive rate of exposed infants were estimated for only 3 low income regions from 2005–2012; the mean estimated rate in these regions for 2005 was 11.8% (95% CI = 0–27.1) compared with 6.1% (95% CI = 2.7–9.4)in 2010. The estimated percentages of initiating ART in HIV-positive women and initiating ARP in exposed infants were continually increased, and the mean estimated percentage of initiating ART in HIV-positive women increased from 64.6% (95% CI = 60.7–68.6)in 2005 to 79.5% (95% CI = 76.0–83.1)in 2012, while initiating ARP in exposed infants increased from 77.2%(95% CI = 73.6–80.7)in 2005 to 91.6%(95% CI = 87.5–95.6) in 2010. The estimated percentage of voluntary HIV-testing and HIV diagnosis of the exposed infants has continually increased to 100% since 2004, while voluntary HIV-counseling has not obviously changed. Interestingly, the estimated percentage of selecting termination of pregnancy decreased from 40% (95% CI = 9.6–70.4)in 2003 to 15.8%(95% CI = 0–32.2)in 2011; the estimated percentage of artificial feeding was not obviously changed.

**Table 3:** Subgroup Analyses of Uptake of Prevention of Mother-to-Child-Transmission (PMTCT) from 2004 to 2007.

Outcomes	Before 2004		2004		2005		2006		2007	
	Studies	Rate(95%CI)	Studies	Rate(95%CI)	Studies	Rate(95%CI)	Studies	Rate(95%CI)	Studies	Rate(95%CI)
HIV-positive rate of pregnant women **(%)**	12	0.1(0.00–0.1)	8	0.10(0.06–0.14)	13	0.01(0.01–0.02)	15	0.11(0.09–0.14)	14	0.12(0.10–0.14)
HIV-positive rate of exposed infants	-	-	-	-	1	0.12(0–0.27)	3	0.08(0–0.17)	3	0.13(0.05–0.22)
Voluntary HIV-counseling	-	-	1	0.81(0.79–0.82)	2	0.81(0.59–1.00)	2	0.9(0.79–1.00)	2	0.88(0.82–0.93)
Voluntary HIV-testing	-	-	5	0.58(0.21–0.95)	8	0.63(0.48–0.78)	9	0.72(0.65–0.79)	9	0.81(0.76–0.85)
Initiating ART in HIV-positive women	-	-	-	-	1	0.65(0.61–0.69)	1	0.67(0.64–0.70)	1	0.67(0.64–0.69)
Initiating ARP in exposed infants	-	-	-	-	1	0.77(0.74–0.81)	1	0.80(0.78–0.83)	1	0.84(0.82–0.86)
HIV diagnosis of the exposed infants	-	-	-	-	1	0.81(0.64–0.98)	3	0.86(0.82–0.89)	3	0.76(0.73–0.79)
Selecting termination of pregnancy	2	0.4(0.10–0.70)	3	0.25(0.07–0.43)	8	0.28(0.22–0.34)	7	0.37(0.30–0.45)	6	0.36(0.27–0.44)
Cesarean section	-	-	-	-	-	-	-	-	-	-
Artificial feeding	-	-	-	-	2	0.87(0.85–0.90)	2	0.82(0.58–1.00)	2	0.92(0.90–0.93)

Note: ARP = antiretroviral prophylaxis; ART = antiretroviral therapy

**Table 4:** Subgroup Analyses of Uptake of Prevention of Mother-to-Child-Transmission (PMTCT) from 2008 to 2012.

Outcomes	2008		2009		2010		2011		2012	
	Studies	Rate(95%CI)	Studies	Rate(95%CI)	Studies	Rate(95%CI)	Studies	Rate(95%CI)	Studies	Rate(95%CI)
HIV-positive rate of pregnant women **(%)**	14	0.10(0.08–0.12)	13	0.07(0.01–0.12)	13	0.09(0.07–0.10)	5	0.10(0.06–0.14)	3	0.15(0.01 to 0.28)
HIV-positive rate of exposed infants	3	0.06(0.01–0.10)	2	0.04(0.00–0.08)	2	0.04(0.00–0.08)	2	0.03(0.00–0.05)	2	0.06(0.03–0.09)
Voluntary HIV-counseling	3	0.88(0.76–0.99)	3	0.80(0.76–0.84)	2	0.64(0.64–0.65)	1	0.83(0.83–0.84)	-	-
Voluntary HIV-testing	10	0.76(0.71–0.82)	8	0.77(0.71–0.83)	6	0.81(0.68–0.93)	4	0.95(0.90–0.99)	3	0.99(0.97–1.00)
Initiating ART in HIV-positive women	1	0.74(0.72–0.77)	2	0.71(0.52–0.90)	4	0.79(0.72–0.86)	1	0.75(0.64–0.86)	1	0.80(0.76–0.83)
Initiating ARP in exposed infants	2	0.83(0.69–0.97)	1	0.83(0.81–0.84)	2	0.92(0.88–0.96)	-	-	-	-
HIV diagnosis of the exposed infants	3	0.74(0.72–0.77)	2	0.73(0.70–0.76)	1	0.82(0.75–0.89)	1	0.99(0.98–1.00)	1	0.98(0.95–1.00)
Selecting termination of pregnancy	6	0.31(0.22–0.41)	4	0.24(0.14–0.35)	3	0.19(0.09–0.28)	1	0.16(0–0.32)	-	-
Cesarean section	-	-	-	-	-	-	-	-	-	-
Artificial feeding	3	0.91(0.84–0.99)	1	0.94(0.93–0.95)	1	0.81(0.73–0.89)	-	-	-	-

Note: ARP = antiretroviral prophylaxis; ART = antiretroviral therapy

The uptake of PMTCT programs varied widely between regions (Table 5). The region with the highest HIV-positive rate of pregnant women was the Xinjiang Uygur [Uighur] autonomous region (0.41%, 95% CI = 0.38–0.44) in northwest China, but only one study included that region in its estimate. The rates in eastern China (0.02%, 95% CI = 0.01–0.02) and south-central China (0.06%, 95% CI = 0.05–0.07) were all less than 0.1% (Table 4). At a provincial level, the estimated voluntary HIV-testing rate ranged from approximately 44.3% (95% CI = 20.4–68.2) in several northwest regions to 79.0%(95% CI = 75.3–82.4)in both south-central China and southwestern China (Table 4). Northwest China also reported the lowest percentage of voluntary HIV-counseling; the estimated mean rate was 19.8% (95% CI = 16.2%-23.5). The rates of initiating ART in HIV-positive women were the highest in northwest China (76.8%, 95% CI = 74.5–79.1), which was followed by south-central China (70.0%, 95% CI = 60.5–77.7); the lowest rates were for eastern China (49.3%, 95% CI = 19.5–79.1) (Table 5).

**Table 5:** Subgroup Analyses of Uptake of Prevention of Mother-to-Child-Transmission (PMTCT) by Region.

Outcomes	Eastern China		South-central China		Northwest China		Southwestern China	
	Studies	Rate(95%CI)	Studies	Rate(95%CI)	Studies	Rate(95%CI)	Studies	Rate(95%CI)
HIV-positive rate of pregnant women(%)	2	0.02(0.01–0.02)	16	0.06(0.05–0.07)	1	0.41(0.39–0.44)	14	0.24(0.20–0.28)
HIV-positive rate of exposed infants	0	-	5	0.04(0.02–0.05)	1	0.06(0.04–0.08)	7	0.04(0.02–0.07)
Voluntary HIV-counseling	3	0.84(0.72–0.97)	1	0.73(0.73–0.74)	1	0.20(0.16–0.24)	5	0.77(0.72–0.82)
Voluntary HIV-testing	3	0.72(0.51–0.93)	7	0.79(0.71–0.87)	3	0.44(0.20–0.68)	11	0.79(0.75–0.82)
Initiating ART in HIV-positive women	2	0.49(0.20–0.79)	13	0.70(0.56–0.84)	1	0.77(0.75–0.79)	13	0.69(0.61–0.78)
Initiating ARP in exposed infants	2	0.66(0.50–0.82)	14	0.78(0.68–0.87)	1	0.83(0.80–0.85)	11	0.75(0.66–0.84)
HIV diagnosis of the exposed infants	0	-	7	0.67(0.53–0.81)	1	0.87(0.84–0.89)	6	0.69(0.38–1.00)
Selecting termination of pregnancy	1	0.43(0.35–0.51)	20	0.41(0.32–0.50)	0	-	12	0.31(0.21–0.42)
Cesarean section	0	-	6	0.54(0.32–0.75)	0	-	5	0.45(0.10–0.79)
Artificial feeding	1	-	12	0.91(0.87–0.94)	0	-	8	0.94(0.92–0.96)

Note: ARP = antiretroviral prophylaxis; ART = antiretroviral therapy

The estimated HIV-positive rate of pregnant women was the highest in studies from low income regions(0.20%, 95%CI = 0.17–0.22), which was followed by low-middle income regions(0.15%, 95%CI = 0.07–0.29). Studies from high-middle income and high income regions reported the lowest prevalence(Table 5). The high income region reported the highest percentage of voluntary HIV-counseling (93.2%, 95%CI = 93.2–93.2), voluntary HIV-testing (94.9%, 95%CI = 88.8–1.00) and cesarean section (81.1%, 95%CI = 70.6–91.7), while the low income region reported the highest percentage of initiating ART in HIV-positive women (79.9%, 95%CI = 75.5–84.3) and initiating ARP in exposed infants (84.6%, 95%CI = 80.8–88.4) (Table 6).

**Table 6:** Subgroup Analyses of Uptake of Prevention of Mother-to-Child-Transmission (PMTCT) by Income.

Outcomes	High income		High-middle income		Low-middle income		Low income	
	Studies	Rate(95%CI)	Studies	Rate(95%CI)	Studies	Rate(95%CI)	Studies	Rate(95%CI)
HIV-positive rate of pregnant women **(%)**	2	0.02(0.02–0.02)	6	0.02(0.01–0.02)	3	0.15(0.07–0.29)	23	0.20(0.17–0.22)
HIV-positive rate of exposed infants	0	-	2	-	1	0.06(0.04–0.08)	9	0.04(0.03–0.05)
Voluntary HIV-counseling	1	0.93(0.93–0.93)	0	-	1	0.89(0.89–0.89)	6	0.86(0.78–0.94)
Voluntary HIV-testing	2	0.95(0.89–1.00)	4	0.95(0.93–0.98)	4	0.64(0.49–0.78)	12	0.87(0.83–0.91)
Initiating ART in HIV-positive women	3	0.65(0.36–0.93)	4	0.57(0.31–0.83)	3	0.61(0.50–0.72)	21	0.80(0.76–0.84)
Initiating ARP in exposed infants	2	0.66(0.50–0.82)	5	0.65(0.47–0.83)	3	0.70(0.61–0.80)	20	0.85(0.81–0.88)
HIV diagnosis of the exposed infants	0	-	2	0.66(0.32–1.00)	2	0.81(0.69–0.92)	12	0.68(0.53–0.84)
Selecting termination of pregnancy	2	0.35(0.20–0.51)	7	0.45(0.36–0.53)	3	0.29(0.28–0.31)	25	0.35(0.27–0.43)
Cesarean section	1	0.81(0.71–0.92)	3	0.57(0.43–0.71)	2	0.49(0.48–0.51)	9	0.43(0.25–0.61)
Artificial feeding	1	-	5	0.77(0.59–0.95)	2	0.95(0.90–1.00)	17	0.94(0.92–0.95)

Note: ARP = antiretroviral prophylaxis; ART = antiretroviral therapy

We estimated that 38.6%(95% CI = 34.0–44.1) of women with HIV received ART antenatally, 35.5% (95% CI = 18.7–53.2)received ART in the intrapartum period, and 27.7% (95% CI = 0.00–75.0)received it postnatally; 47.6%(95% CI = 18.9–75.2) received single-dose nevirapine (NVP), 21.6% (95% CI = 9.1–34.8)received single-dose zidovudine (AZT), 18.9% (95% CI = 8.9–28.4)received combination of AZT and NVP, 13.4% (95% CI = 2.4–25.3)received combination of AZT and lamivudine (3TC), and 31.3%(95% CI = 16.2–47.0) received combination of three antiretroviral drugs (AZT+3TC/AZT+NVP). In addition, 46.6% (95% CI = 7.1–85.8)of exposed infants received single-dose NVP, 18.9% (95% CI = 1.0–36.3)received single-dose AZT, 53.3% (95% CI = 40.1–66.7)received a combination of AZT and NVP, and fewer than 1% (95% CI = 0.0–3.0) received the combination of three antiretroviral drugs.

### Sensitivity Analyses

We used the fixed effect model and trim and fill analysis, and we excluded studies with fewer participants to perform sensitivity analyses of the uptake of PMTCT programs, which gave similar results to the primary analysis.

### Meta-regression analysis, assessment of publication bias

We noted significant heterogeneity within studies(P<0.001, I^2^ = 95.1%–100%) except for the outcome of estimated HIV-positive rate of exposed infants (P = 0.602, I^2^ = 7.1). In univariate and multivariable meta-regression analyses (Table 6), we usedvariables including year of publication, sample size, regions (eastern China, south-central China, northwest China, southwestern China, and trans-regional), income level, and quality score. We notedthat regions, income level, and sample size were significantly associated with the estimated HIV-positive rate of pregnant women; year of publication was significantly associated with the percentage of voluntary HIV-testing (R^2^ = 25.35%, P = 0.004); year of publication (R^2^ = 30.63%, P = 0.056) and sample size (R^2^ = 38.68%, P = 0.032) were significantly associated with the percentage of voluntary HIV-counseling; income level were significantly associated with the percentage of initiating ARP in exposed infants (R^2^ = 12.36%, P = 0.043); year of publication was significantly associated with the percentage of selecting termination of pregnancy (R^2^ = 17.59%, P = 0.007). Furthermore, in multivariable analysis these variables still were significantly associated with the heterogeneity of these main outcomes (Table 6).

Egger’slinear regression test (P = 0.093) and Begg’s test (P = 0.204) shown significantpublication bias among the contributing studies in terms of the outcome of the estimated HIV-positive rate of pregnant women, the percentage of initiating ART in HIV-positive women, the percentage of initiating ARP in exposed infants, and the percentage of artificial feeding (Table 7).

**Table 7:** Results of Meta-regression for prevention of mother-to-child-transmission (PMTCT) of China.

Covariate	Univariate analyses			Multivariable analyses		
	Coefficient (95% CI)	P value	Variance explained (%)	Coefficient (95% CI)	P value	Variance explained (%)
Estimated HIV-positive rate of pregnant women						37.03
Year of publication	-0.0001(-0.0006 to 0.0003)	0.553	-2.43			
Regions (middle south is reference)			28.59			
Eastern China	-0.0005(-0.0034 to 0.0023)	0.701		0.0004(-0.0025 to 0.0034)	0.771	
Northwest China	0.0034(-0.0012 to 0.0081)	0.147		0.0041 (-0.0004 to 0.0085)	0.073	
Southwestern China	0.0029(0.0011 to 0.0046)	0.002		0.0021(0.0004 to 0.0085)	0.002	
Income level	-0.0010(-0.0019 to-0.0001)	0.032	11.51	-0.0003(-0.0013 to 0.0006)	0.457	
Sample size (<100000 vs ≥100000)	-0.0025(-0.0042 to-0.0009)	0.004	21.47	-0.0017(-0.0034 to-0.0000)	0.044	
Quality score	-0.0002 (-0.0008 to 0.0005)	0.625	-2.59			
Percentage of voluntary HIV-counseling						-17
Year of publication	-0.014(-0.090 to 0.063)	0.705	-5.53	0.219(-0.080 to 0.518)	0.199	
Regions (middle south is reference)			-14.92			
Northwest China	0.211(-0.494 to 0.916)	0.523		0.488(-0.749 to 1.724)	0.382	
Southwestern China	0.034(-0.340 to 0.408)	0.844		0.597(-0.342 to 1.536)	0.176	
Income level	0.005(-0.214 to 0.223)	0.964	-0.735	0.248(-0.199 to 0.695)	0.232	
Sample size (<100 vs ≥100)	0.093(-0.059 to 0.245)	0.211	3.95	0.219(-0.080 to 0.518)	0.127	
Quality score	-0.033(-0.155 to 0.089)	0.571	-4.17	0.054 (-0.164 to 0.272)	0.578	
Percentage of voluntary HIV-testing						29.54
Year of publication	0.056(0.019 to 0.093)	0.004	25.35	0.05(0.009 to 0.091)	0.02	
Regions (middle south is reference)			6.41			
Northwest China	-0.334(-0.725 to 0.057)	0.091		-0.282(-0.635 to 0.07)	0.11	
Southwestern China	-0.092(-0.328 to 0.144)	0.427		0.046(-0.195 to 0.287)	0.694	
Eastern China	-0.198(-0.498 to 0.102)	0.186		-0.193(-0.466 to 0.079)	0.153	
Trans-reginoal	0.096(-0.237 to 0.429)	0.554		-0.037(-0.357 to 0.284)	0.812	
Income level	0.049(-0.05 to 0.148)	0.318	0.11	-0.021(-0.132 to 0.09)	0.694	
Sample size (<100 vs ≥100)	0.153(-0.038 to 0.345)	0.112	6.14	0.149(-0.074 to 0.372)	0.177	
Quality score	0.041(-0.029 to 0.111)	0.239	1.76	0.035(-0.038 to 0.107)	0.327	
Percentage of voluntary HIV-counseling						99.06
Year of publication	0.075(-0.003 to 0.153)	0.056	30.63	0.034(-0.233 to 0.302)	0.35	
Regions (middle south is reference)			-11.09			
Northwest China	-0.535(-1.718 to 0.649)	0.298		-0.912(-1.754 to-0.07)	0.046	
Southwestern China	0.025(-0.891 to 0.941)	0.947		-0.664(-1.395 to 0.067)	0.055	
Eastern China	0.112(-0.912 to 1.136)	0.79		-0.882(-1.84 to 0.075)	0.054	
Trans-reginoal	0.122(-1.06 to 1.305)	0.801		-0.205(-0.809 to 0.4)	0.145	
Income level	0.027(-0.231 to 0.285)	0.818	-11.77	0.327(0.048 to 0.606)	0.043	
Sample size (<10000 vs ≥10000)	0.431(0.046 to 0.815)	0.032	38.68	0.647(-0.534 to 1.828)	0.091	
Quality score	0.016(-0.182 to 0.214)	0.857	-12.08	-0.418(-0.71 to-0.125)	0.035	
Percentage of initiating ART in HIV-positive women						8.65
Year of publication	0.01(-0.023 to 0.043)	0.554	-2.22	0.043(0.001 to 0.085)	0.043	
Regions (middle south is reference)			-8.48			
Northwest China	0.083(-0.354 to 0.519)	0.701		0.041(-0.381 to 0.462)	0.844	
Southwestern China	0.009(-0.166 to 0.184)	0.918		-0.095(-0.274 to 0.083)	0.283	
Eastern China	-0.057(-0.288 to 0.174)	0.618		0.01(-0.244 to 0.265)	0.933	
Trans-reginoal	0.104(-0.125 to 0.332)	0.361		0.139(-0.088 to 0.366)	0.22	
Income level	-0.063(-0.129 to 0.003)	0.06	-7.18	-0.091(-0.178 to-0.003)	0.042	
Sample size (<100 vs ≥100)	-0.064(-0.204 to 0.075)	0.353	-0.02	-0.112(-0.273 to 0.049)	0.166	
Quality score	-0.015(-0.069 to 0.038)	0.563	-2.00	-0.02(-0.087 to 0.046)	0.533	
Percentage of initiating ARP in exposed infants						-11.63
Year of publication	0.005(-0.036 to 0.046)	0.815	3.94	0.002(-0.053 to 0.058)	0.934	
Regions (middle south is reference)			-10.39			
Northwest China	0.07(-0.353 to 0.493)	0.736		0.058(-0.416 to 0.532)	0.801	
Southwestern China	-0.009(-0.188 to 0.169)	0.915		-0.073(-0.29 to 0.145)	0.492	
Eastern China	-0.117(-0.444 to 0.209)	0.465		0.115(-0.307 to 0.536)	0.577	
Trans-reginoal	0.121(-0.142 to 0.384)	0.351		0.047(-0.257 to 0.35)	0.752	
Income level	-0.074(-0.145 to-0.002)	0.043	12.36	-0.108(-0.227 to 0.012)	0.075	
Sample size (<100 vs ≥100)	0.067(-0.087 to 0.222)	0.381	-1.3	0.047(-0.146 to 0.24)	0.616	
Quality score	-0.003(-0.067 to 0.061)	0.93	-4.2	-0.003(-0.089 to 0.083)	0.935	
Percentage of selecting termination of pregnancy						23.18
Year of publication	-0.039(-0.066 to-0.011)	0.007	17.59	-0.05(-0.082 to-0.019)	0.003	
Regions (middle south is reference)						
Southwestern China	-0.098(-0.242 to 0.047)	0.179	-1.37	-0.042(-0.181 to 0.097)	0.54	
Eastern China	-0.061(-0.354 to 0.232)	0.674		-0.141(-0.459 to 0.178)	0.373	
Trans-reginoal	-0.138(-0.377 to 0.102)	0.251		-0.164(-0.385 to 0.058)	0.141	
Income level	0.023(-0.044 to 0.091)	0.482	-1.35	0.057(-0.023 to 0.138)	0.156	
Sample size (<100 vs ≥100)	-0.1(-0.226 to 0.025)	0.114	4.43	-0.037(-0.165 to 0.091)	0.557	
Quality score	0.009(-0.04 to 0.058)	0.718	-2.42	0.013(-0.035 to 0.061)	0.583	
Percentage of cesarean section						-41.07
Year of publication	0.02(-0.052 to 0.092)	0.555	-4.47	0.018(-0.106 to 0.143)	0.743	
Regions (middle south is reference)			-2.37			
Southwestern China	-0.088(-0.443 to 0.267)	0.599		0.199(-0.748 to 1.146)	0.641	
Eastern China	0.276(-0.358 to 0.911)	0.361		-0.101(-1.436 to 1.234)	0.866	
Trans-reginoal	-0.176(-0.552 to 0.199)	0.327		0.208(-0.929 to 1.346)	0.684	
Income level	0.096(-0.044 to 0.237)	0.162	7.96	0.248(-0.465 to 0.961)	0.446	
Sample size (<100 vs ≥100)	-0.149(-0.434 to 0.135)	0.279	2.1	-0.163(-0.76 to 0.434)	0.546	
Quality score	0.016(-0.091 to 0.122)	0.758	-6.42	-0.104(-0.432 to 0.224)	0.486	
Percentage of artificial feeding						-11.37
Year of publication	-0.011(-0.033 to 0.01)	0.295	-0.03	0.016(0.022 to 0)	-0.046	
Regions (middle south is reference)			-20.67			
Southwestern China	0.042(-0.049 to 0.132)	0.346		0.033(-0.078 to 0.144)	0.534	
Trans-reginoal	0.017(-0.128 to 0.161)	0.813		-0.055(-0.217 to 0.106)	0.476	
Income level	-0.049(-0.104 to 0.007)	0.081	8.9	-0.042(-0.12 to 0.036)	0.267	
Sample size (<100 vs ≥100)	0.047(-0.029 to 0.122)	0.21	2.68	0.079(-0.013 to 0.171)	0.088	
Quality score	-0.012(-0.043 to 0.019)	0.416	-2.27	0.009(-0.03 to 0.048)	0.644	

## Discussion

This is the first comprehensive overview of PMTCT programs in China at the national level. We included 57 studies covering all provinces in China, and there was no report on PMTCT programs before 2004, which may be because the Chinese ministry of health first issued guidelines on PMCTC in 2004[6].Our review showed that the overall uptake of PMTCT programs was low and did not reach the 80% target that was setby the United Nations General Assembly Special Session(UNGASS)[74]. The estimated percentage of antiretroviral therapy in HIV-positive pregnant women was still unsatisfactory. We estimated that the MTCT rate in China was 4.4% (95% CI, 3.2–5.5), which was substantially higher than in the U.S. and Europe (less than 1%), while it was lower than some low- and middle-income countries in Sub-Saharan Africa (11%)[1,75]. Great successes in reducing the MTCT rate has been achieved in China (reduced from 11.8% in 2005 to 4.2% in 2009) due to the scale up of PMTCT programs since the Ministry of Health launched PMTCT programs in 2003. In 2001 UNGASS set a goal that would reduce theproportion of HIV infected infants by 50% in ten years, and in order to achieve this target they estimated that 80% of pregnant women andtheir children need to receive PMTCT programs service [74]. Despite efforts to increase the uptake of PMTCT interventions services, coverage is still lower than desired in China. Many international non-governmental organizations, such as the UN Secretary General, G8 countries, the Global Fund toFight AIDS, WHO and so on have committed to further develop and improve the quality and effectiveness ofPMTCT service coverage in low- and middle-income countries[75]. Integration of PMTCT with other healthcare services, such as maternal, newborn, and child health may be a crucialcomponent of the strategy to scale up PMTCT programs. In China, PMTCT services were integrated with antenatal care and perinatal care, pregnant women were provided with HIV testing and counseling and HIV positivepregnantwomen were provided with antiretroviral prophylaxis in antenatal care and attending labor ward.Discouragingly, a recent review noted very limited, non-generalizableevidence of improved PMTCT intervention uptake in integratedPMTCT programscompared to non- or partially integrated services[76].A reviewassessing the role of family planning in eliminating new pediatric HIV infectionsreported that integrating family planning and HIV services is an effective strategy for increasing access to contraception among women with HIV who do not wish to become pregnant, which could accelerate the ending of new pediatric HIV infections[77]. In recent years, PMTCT servicewas also integrated with family planning service in China; HIV testing was provided forwomen of childbearing age when attending national free pre-pregnancy eugenic health check project, and the HIV-positive women were suggested that they should receive antiretroviral therapy before pregnancy or prevent unintended pregnancies by the use of contraception. But the coverage of integrated family planning/HIV service was very low in China, which prompt the Chinese government to call for increaseddomestic and international financing to expand the uptake of PMTCT.

We noted significant heterogeneity within studies in this meta-analysis, and meta-regression analyses revealed that year of publication was the main factors that explained much of the heterogeneity between studies.PMTCT programs have changed significantly over the years, and there are guidelines for starting ART for both women and infants. While this is acknowledged, treating all studies from 2000 to 2014 as equivalent leads to very heterogeneous data. For example, if the risk of MTCT was high in 2000, when presumably no ART was administered, it is unfair to combine these data with recent guidelines to provide ART if CD4<500 or lifelong ART (Option B+). Therefore, we performed subgroup analysis by years and present the historical and current policies for PMTCT services.Study region and income level may also account for much of the heterogeneity between studies. For example, northwest China had the highest MTCT rate (5.9%)and lowest uptake of PMTCT programs services; the high income region usually reported the highest percentages of voluntary HIV-counseling (93.2%) and voluntary HIV-testing (94.9%).The variation between regions and income levels were consistent with the global uptake of HIV testing; the low income countries are reported to have low uptake of HIV testing, and the coverage for early infant diagnosis of HIV was below 6% in some low income countries (Angola, Nigeria, Malawi, Democratic Republic of Congo, and Chad) in 2012[78,79].Such variation could be explained by lower government financial investment, limited health resources and inefficient programs implementation strategy in regions with low uptake of PMTCT services.Voluntary counseling and testing (VCT) was effective in reducing MTCT as well as cost effective as a PMTCT intervention. The WHO had recommend that all children younger than 2 years old who are living with HIV should be treated, and the important first step was to identify the HIV-infected children through developing effective strategies for HIV testing. Although HIV testing facilities have increased over the past ten years, the uptake of VCT has still been low; UNAIDS reported in 2012 that 50% of individuals living with HIV were unaware of their HIV status[80]. Encouraging data are presented in this review, including that the estimated percentage of voluntary HIV-testing in pregnant women continually increased from 57.9% in 2004 to 98.5% in 2012 and that the estimated infant free HIV antibody testing rate over 18 months of age increased from 81% in 2005 to 97.7% in 2012. The number of new HIV-infected children in the 21 priority African countries in the UN Programme on HIV/AIDS (UNAIDS) global plan decreased by 38% between 2009 and 2012 because of increased access to antiretroviral treatment to prevent MTCT[81,82]. We estimated that more than 70% of HIV-infected pregnant women never received antiretroviral treatmentin antenatal care in China, which is significant lower than that in low- and middle- income countries with only 55% (range 22–99%) of HIV positive women starting highly active antiretroviral therapy in antenatal care[75,83]. An encouraging result in this meta-analysis was that initiating ARP in exposed infants continually increased from 77.0% in 2005 to 98.1% in 2012, which achieved the goal of the 12thFive-Year action plan on containment, prevention and control of HIV/AIDS that the percentage of HIV exposed infants who received ARV would be more than 90% by the year 2015[84]. According to recently WHO report, the use of combination ART during pregnancy is preferable to single therapy[85]. The combination ART is more effective at PMTCT, and it has the advantages of reducing sexual HIV transmission and HIV-associated morbidity and mortality[86]. In China, we estimated that approximately 31.3% (95% CI, 15.5–47.0) of women with HIV received the combination of three antiretroviral drugs (AZT+3TC/AZT+NVP), and < 1% of exposed infants received the combination of three antiretroviral drugs.

Despite the great successes in reducing MTCT in China, we are facing many challenges, such as low coverage of PMTCT programs, stigma and discrimination, drug resistance, and delayed infant HIV diagnosis[5]. This review had several limitations; firstly, significant heterogeneity between studies was observed. Although we performed subgroup analyses by publication year, geographical area, and income level, and these factors may be the sources of between-study heterogeneity. However other unmeasured characteristics in study population and limitations of the included studies likely influence the detected heterogeneity; unfortunately, we did not obtain enough information about these aspects for further analysis. Secondly, we have only conducted the search in electronic databases. Studies published in local journals which are not indexed in electronic databases might have been missed out in this review. The third, results from Begg’s funnel plot and Egger’s test are different but the funnel plot and Trim and Fill methods suggested the presence of a potential publication bias, a language bias, and inflated estimates by a flawed methodological design in smaller studies.The last, some pooled estimates in subgroups should be interpreted with caution due the small number studies.

In conclusion, PMTCT programs have scaled up quickly in recent years in China. However, antiretroviral therapy in HIV-positive pregnant, antiretroviral prevention and HIV diagnosis in exposed infants were still unsatisfactory. Moreover, there was a big gap of uptake of PMTCT programs between regions and income levels. These results highlight the need to further scale up PMTCT services, especially in regions with the lowest coverage, so that more women can access and utilize them.

## Supporting Information

S1 PRISMA Checklist PRISMA-2009- checklist. (DOC)

S1 Appendix Search Terms of the Meta-analysis. Search terms consisted of the following key wordsincludingPMTCT, HIV, and China were provided.
(DOCX)
