# Supplementary material for: The Uptake of Prevention of Mother-to-Child HIV Transmission Programs in China: A Systematic Review and Meta-Analysis
Source: PLoS One. 2015 Aug 26;10(8):e0135068. doi: 10.1371/journal.pone.0135068 (PMC4550389; doi:10.1371/journal.pone.0135068)
Supplement: S1 Appendix — Search terms consisted of the following key wordsincludingPMTCT, HIV, and China were provided. (DOCX) [file pone.0135068.s002.docx]

Appendix 1. Search terms of the meta-analysis

Search terms consisted of the following key words: PMTCT (mother-to-child transmission, vertical transmission, vertical infectious diseases transmission, fetomaternal infection transmission, PMTCT, MTCT); HIV (HIV, human immune deficiency virus, human immunodeficiency virus, human immuno-deficiency virus, human immune*, acquired immunodeficiency syndrome, acquired immuno-deficiency syndrome, acquired immunodeficiency syndrome, acquired immune-deficiency syndrome, acquired immune*); and China (China, Chinese, CN, CHN, Mongolia, Sinkiang, Tibet, Far East, Asia).
